# DNA Copy Number Alterations and Copy Neutral Loss of Heterozygosity in Adult Ph-Negative Acute B-Lymphoblastic Leukemia: Focus on the Genes Involved

**DOI:** 10.3390/ijms242417602

**Published:** 2023-12-18

**Authors:** Natalya Risinskaya, Maria Gladysheva, Abdulpatakh Abdulpatakhov, Yulia Chabaeva, Valeriya Surimova, Olga Aleshina, Anna Yushkova, Olga Dubova, Nikolay Kapranov, Irina Galtseva, Sergey Kulikov, Tatiana Obukhova, Andrey Sudarikov, Elena Parovichnikova

**Affiliations:** 1 National Medical Research Center for Hematology, 125167 Moscow, Russia; makislitsyna@gmail.com (M.G.); patakh1997@mail.ru (A.A.); uchabaeva@gmail.com (Y.C.); surimova.lera@mail.ru (V.S.); dr.gavrilina@mail.ru (O.A.); ann.unikova@bk.ru (A.Y.); dubovaolgaa@gmail.com (O.D.); immunophenotyping.lab@gmail.com (N.K.); irinagaltseva@gmail.com (I.G.); smkulikov@mail.ru (S.K.); dusha@blood.ru (A.S.); parovichnikova.e@blood.ru (E.P.); 2 Institute of Biodesign and Modeling of Complex Systems, I.M. Sechenov First Moscow State Medical University, 119991 Moscow, Russia

**Keywords:** acute B-lymphoblastic leukemia (B-ALL), copy number alterations (CNAs), copy neutral loss of heterozygosity (cnLOH), molecular karyotype, genes

## Abstract

The landscape of chromosomal aberrations in the tumor cells of the patients with B-ALL is diverse and can influence the outcome of the disease. Molecular karyotyping at the onset of the disease using chromosomal microarray (CMA) is advisable to identify additional molecular factors associated with the prognosis of the disease. Molecular karyotyping data for 36 patients with Ph-negative B-ALL who received therapy according to the ALL-2016 protocol are presented. We analyzed copy number alterations and their prognostic significance for *CDKN2A*/*B*, *DMRTA*, *DOCK8*, *TP53*, *SMARCA2*, *PAX5*, *XPA*, *FOXE1*, *HEMGN*, *USP45*, *RUNX1*, *NF1*, *IGF2BP1*, *ERG*, *TMPRSS2*, *CRLF2*, *FGFR3*, *FLNB*, *IKZF1*, *RUNX2*, *ARID1B*, *CIP2A*, *PIK3CA*, *ATM*, *RB1*, *BIRC3*, *MYC*, *IKZF3*, *ETV6*, *ZNF384*, *PTPRJ*, *CCL20*, *PAX3*, *MTCH2*, *TCF3*, *IKZF2*, *BTG1*, *BTG2*, *RAG1*, *RAG2*, *ELK3*, *SH2B3*, *EP300*, *MAP2K2*, *EBI3*, *MEF2D*, *MEF2C*, *CEBPA*, and *TBLXR1* genes, choosing t(4;11) and t(7;14) as reference events. Of the 36 patients, only 5 (13.8%) had a normal molecular karyotype, and 31 (86.2%) were found to have various molecular karyotype abnormalities—104 deletions, 90 duplications or amplifications, 29 cases of cnLOH and 7 biallelic/homozygous deletions. We found that 11q22-23 duplication involving the *BIRC3*, *ATM* and *MLL* genes was the most adverse prognostic event in the study cohort.

## 1. Introduction

B-cell acute lymphoblastic leukemia is a malignant neoplasm characterized by proliferation of lymphoid cell precursors, leading to infiltration of the bone marrow by lymphoblasts. Over the past 5 years, significant progress has been achieved in the treatment and molecular diagnosis of B-ALL. According to Parovichnikova et al. [1], two laboratory parameters remain associated with poor disease outcomes. Any abnormal cytogenetic karyotype, excluding hyperploidy, and measurable residual disease (MRD) positivity on day +70 (after completion of two phases induction therapy) are considered as risk factors for Ph-negative B-cell ALL. The identification of certain prognostic factors is directly related to the choice of therapeutic tactics and the need for allogeneic hematopoietic stem cell transplantation (allo-HSCT) after achieving the first complete remission. To detect chromosomal abnormalities and identify risk groups, traditional cytogenetic analysis (CCA, conventional chromosomal analysis) and fluorescent in situ hybridization (FISH) are used. However, in rare cases, due to the absence or insufficient quantity of mitotic cells in the sample, standard cytogenetic testing is not applicable. Moreover, the above methods are not able to detect copy neutral loss of heterozygosity (cnLOH), which leads to the loss of alleles of genes included in the aberration region, while maintaining the number of DNA copies [2]. CnLOH occurs due to events of mitotic homologous recombination as an attempt to correct deletions of chromosomal material utilizing the remaining alleles as a template or is a consequence of mitotic errors, including chromosomal dissegregation [3]. Chromosomal microarray (CMA) can detect cnLOH and DNA copy number alterations as a result of deletions and duplications in the tumor genome. In addition, microdeletions and microduplications from 50 kb to 5 Mb can be detected, and the CMA software (Chromosome Analysis Suite (ChAS 4.3.0.71) in our case) makes it possible to identify a set of genes involved in these aberrations with sufficiently high accuracy. However, CMA does not detect translocations, inversions, or any other aberrations that maintain allelic balance. Therefore, with a joint analysis of cytogenetic and molecular karyotypes, the possibility of the most complete karyotyping appears. We conducted a study of genomic tumor DNA at the onset of the disease in a sample of 36 adult patients with de novo Ph-negative B-ALL. The patients belonged to the same risk group and received treatment according to the same clinical protocol. Therefore, we had an opportunity to assess the associations of the aberrations we found with the features of the disease. The objectives of the study were as follows: comparison of cytogenetic and molecular karyotypes of B-ALL, description of the molecular karyotype features at the onset of the disease in patients with B-ALL and assessment of the prognostic significance of molecular karyotype aberrations.

## 2. Results

The main characteristics of 36 patients included in the study are presented in Table 1.

As a first step, we compared the results obtained using the CMA method with the results of CCA and FISH performed on patients as part of the RALL-2016m protocol at the onset of the disease (Table 2). Aberrations larger than 5 Mb and additionally microdeletions involving *BTG1, CDKN2A/B, EBF1, ERG, ETV6, IKZF1, PAX5*, and *RB1* genes were taken into analysis according to the guidelines for genomic array analysis in acquired haematological neoplastic disorders [4]. Aberrations smaller than 5 Mb should only be accepted for consideration if they involve known leukemia-associated genes or other neoplasia-associated genes, including cell cycle regulators, oncogenes, and tumor suppressor genes. In this exploratory work, we tested most microdeletions, microduplications, and copy neutral LOH sites for the inclusiveness of genes potentially involved in the pathogenesis of B-ALL, and we found potential candidate genes almost everywhere. A complete table with the sizes of the found aberrations is presented in Table S1.

For most patients, the data from CMA and CCA do not contradict each other. Additionally, areas of copy neutral LOH, microdeletions and microduplications that were not detected by standard cytogenetics were identified. However, CMA provides information only about the number of DNA copies and areas of LOH, but not about the structure of the genome and the location of aberrant DNA fragments. There is a logical explanation for the apparent discrepancies, related either to the insufficiency of mitoses in CCA, or to the identification of minor clones by the CMA method.

### 2.1. Clonal Heterogeneity in B-ALL

**Figure 1:**
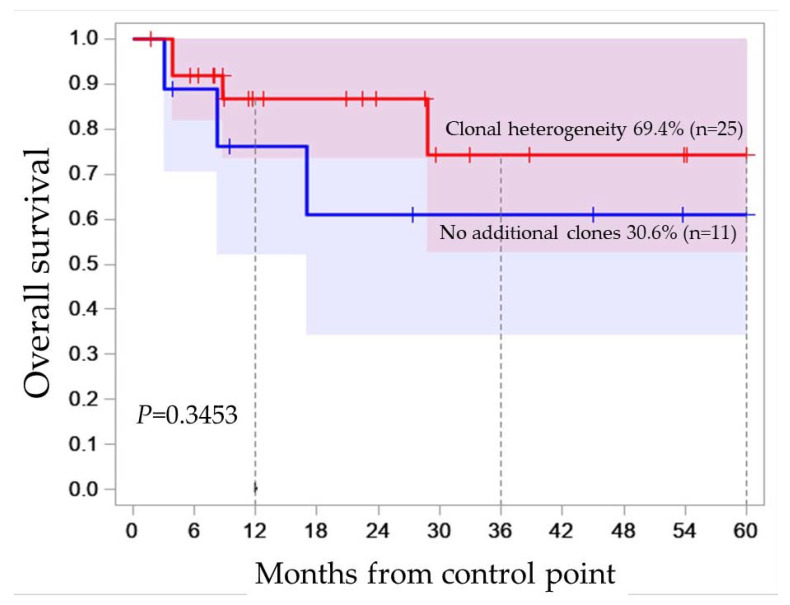
Kaplan–Meier survival curve for OS estimates according to the clonal heterogeneity status, red line—clonal heterogeneity, blue line—no additional clones revealed.

B-ALL is characterized by the phenomenon of clonal heterogeneity, when a patient has two or more tumor clones. For example, in the molecular karyotype of patient N°7 (Xp22.33p11.3×1 [0.2], Xp11.3q28×1 [0.5], 3p26.3p12.2×1 [0.3], 3p12.1p11.1×2 hmz, 3q25.33q29×3 [0.3], 6q16.3q24.3×1 [0.6], (9) ×1 [0.21], (11) ×1 [0.3], 12p13.33p13.2×1 [0.22], 12p13.2p12.1×2 hmz [0.5], 12p11.23q22×3 [0.3], 12q22q24.33×1 [0.2], (15) ×1 [0.72], 17p13.3q21.31×1 [0.2], 17q21.31q25.3×2 hmz [0.5], 21q22.12q22.3×3 [0.3]) we see aberrations with mutant allele burden from 0.72 to 0.2. Obviously, aberrations with a load of 0.2 are present in 20% of genomes, and with a burden of 0.7 in 70%, that is, not all genomes contain both aberrations, which means that the clones differ. However, most minor clones at the onset of the disease cannot be detected by routine methods. In the study cohort, we noticed the presence of additional aberrant clones in 19 of 36 patients. Heterogeneity in the onset was also noted during immunophenotyping; in 12 patients out of all who underwent immunophenotyping, two or more immunophenotypes were noted, for example, in patient N°12 CD19+CD10+/−CD34+CD58highCD20-CD22+/−CD24+CD38+CD45−/+. Moreover, in six patients, heterogeneity was noted only in the repertoire of surface antigens (Table S2). Therefore, to test the hypothesis of whether the presence of additional clones was an independent additional risk factor, we assessed OS as a function of the presence of any additional clones noticed in 25 (19 + 6) from 36 patients. There was no significant association of OS with clonal heterogeneity at disease onset (P_OS_ = 0.3453) (Figure 1).

### 2.2. Molecular Karyotype of B-ALL

**Figure 2:**
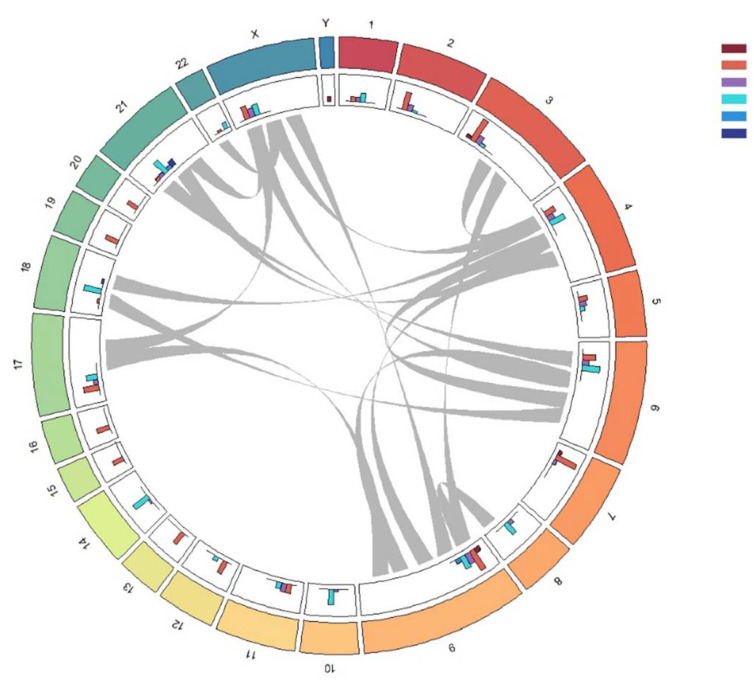
Circos plot for distribution of aberrations in chromosomes. In the outer circle, the size of the segment is proportional to the frequency of aberrations on this chromosome, the second circle comprises histograms with frequencies by copy number, and the inside segments are comparable to each other. Colors represent: dark red—0 copies, red—1 copy, purple—copy neutral LOH, light blue—3 copies, blue—4 copies, dark blue—5 copies of DNA. The gray arches inside indicate the most common combinations, simultaneous alterations in different chromosomes, occurring in more than 20% (8 of 36) of patients.

Only 5 of 36 patients (13.8%) had a normal molecular karyotype, and 31 (86.2%) were found to have various molecular karyotype abnormalities, such as multiple duplications, deletions, biallelic deletions, and cnLOH. The frequencies of occurrence of various types of anomalies on each chromosome and the most common cases of a combination of various anomalies on different chromosomes are presented in Figure 2. The most common anomaly noted was deletion (104 deletions in total), the second most common—duplication/amplification (90 events), and cnLOH (29 cases). Biallelic deletion was detected seven times. We also noted a combination of aberrations in individual patients. Most often, chromosomal abnormalities in combination with other chromosomes were noted for chromosomes 4, 6 and 9 (Figure 2).

### 2.3. Molecular Karyotype Is Not Associated with MRD

**Figure 3:**
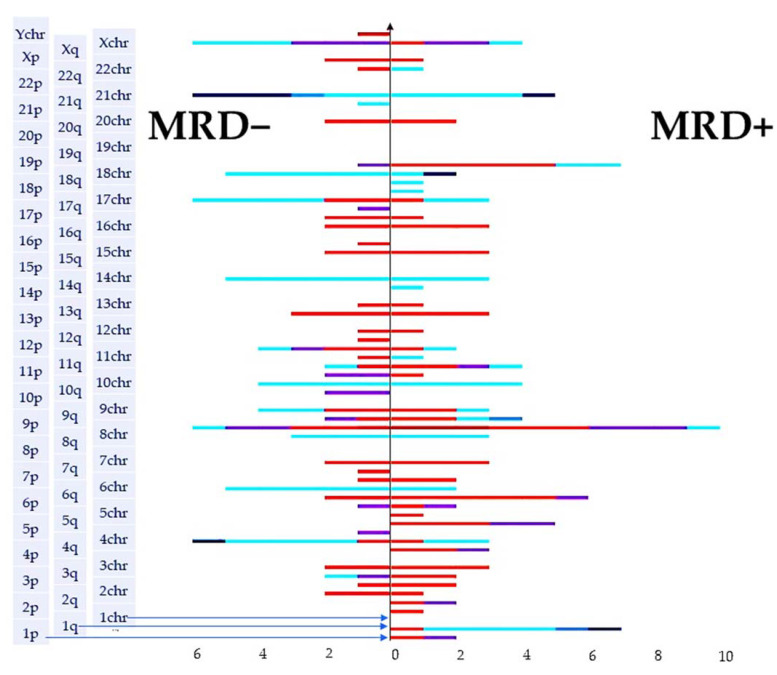
Tornado plot for the distribution of aberrations depending on MRD status. *X*-axis (vertical) is labeled down up “1p, 1q, 1chr” ets. by short and long arms and whole chromosomes numbers from 1to Y. Colors represent: dark red—0 copies, red—1 copy, purple—copy neutral LOH, light blue—duplications, deep blue—amplifications.

From many studies on MRD monitoring in patients with acute leukemia, it is known that the current MRD status is an informative indicator of response to therapy and a significant prognostic factor. We performed a univariate frequency analysis of the association of MRD status at day 70 with the presence of genetic aberrations for each chromosome. We did not identify significant associations between the frequency of achieving MRD-negative status and genetic aberrations, which may be due to the insufficient sample size. Visual analysis of the tornado plot presented in Figure 3 allows us to note that multiple aberrations were present in both groups; however, duplication of 1q, deletions or cnLOH of 5q were found only in the MRD+ group. CnLOH 10q, 11p, deletions of 7q, and Y-chromosome were detected only in the MRD− group.

**Figure 4:**
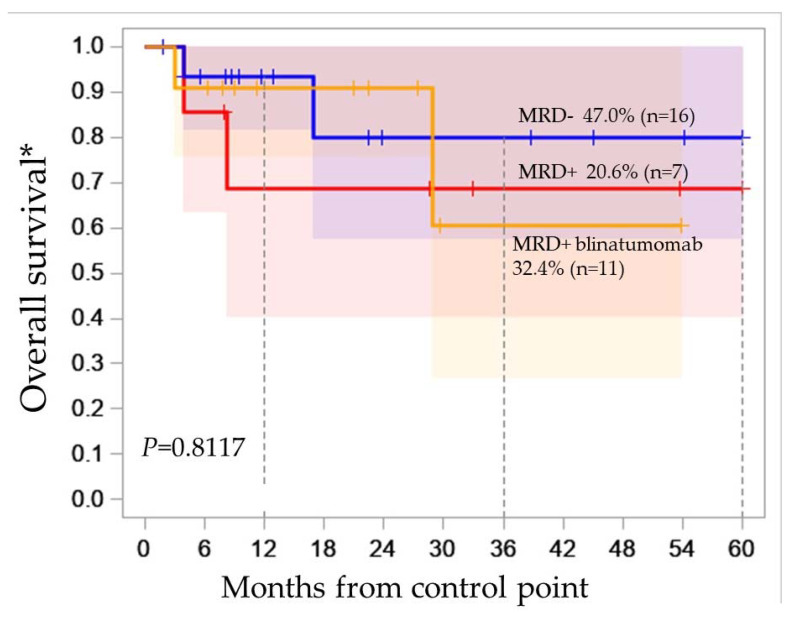
Kaplan–Meier survival curve for OS estimates according to the 70-day MRD status, red line—MRD+, blue line—MRD−, yellow line—MRD+ blinatumomab. * Overall survival was estimated for 34 patients.

We analyzed the association of OS with MRD status at day 70. We did not find significant differences in OS, since the majority of patients with MRD+ status at day 70 received blinatumomab in subsequent cycles of therapy (Figure 4). In group MRD+ without blinatumomab, two patients underwent allo-HSCT. The result of the analysis prompted us to further study the significance of abnormalities in the B-ALL genome without taking into account MRD status.

### 2.4. Loss of Both Alleles—Biallelic or Homozygous Deletion

**Figure 5:**
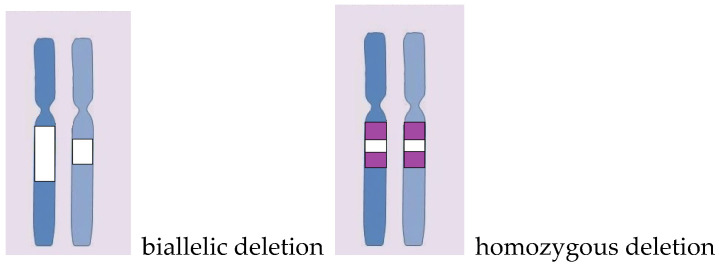
Schematic representation of difference between biallelic deletion and homozygous deletion. On the left—two deletions overlap, on the right—one deletion falling into the region of a cnLOH.

In several patients, we noted a complete loss (copy number 0) of certain DNA loci. These could be due to the overlap of biallelic deletions or the deletion falling into the region of a copy neutral loss of heterozygosity (Figure 5, Table 3).

Four patients had biallelic deletion 9p21.3 (three of them had a deletion under LOH) of the tumor suppressor gene cluster (*CDKN2A*, *CDKN2BAS1*, *CDKN2B*, and *DMRTA1*) at chromosome 9p21. The *CDKN2B-CDKN2A* gene product is a functional RNA molecule that interacts with polycomb repressive complex-1 (PRC1) and -2 (PRC2), leading to epigenetic silencing of other genes in this cluster.

A gene that falls within the region of a microdeletion attracts special attention, allowing for the identification of tumor suppressor factors, such as the loss of the *FLNB* gene in patient 38 (Table 3). Filamin B (FLNB) is known to suppress local tumor growth, angiogenesis, and metastasis [5,6], and its loss may also play a significant role in B-ALL. The copy number of this gene was analyzed in the entire cohort. In eight patients, loss of the *FLNB* allele was detected (22%), in some due to monosomy, in others as part of a deletion of chromosomal fragment. None of the patients had duplications involving this gene. We included this gene in a panel to predict the most adverse genomic events.

### 2.5. HLA-haplotype Loss

We expected to find several patients with HLA-haplotype loss due to 6p or cnLOH deletion. However, only one patient, N°39, showed a copy neutral loss of heterozygosity at onset, which included loss of the HLA haplotype (6p22.2p21.32(25596422_32996007) x2 hmz). The patient had MRD+ status on the day 70 of therapy, a relapse developed 4 months after the start of therapy, and death from the primary disease occurred six months after the start of therapy.

### 2.6. Loss of 3p21.3 Tumor Suppressor Genes Cluster

LOH of 3p21.3—TSG cluster (*CACNA2D2*, *CYB561D2*, *101F6*, *NPRL2*, *ZMYND10*, *RASSF1*, *TUSC2*, *HYAL2* and *HYAL1*) was observed in seven patients. This cluster of tumor suppressors, which was repeatedly studied in solid tumors, attracted our attention when analyzing the molecular karyotype of patient N°15. This patient, with multiple duplications in the genome (Table 2), had a small, slightly more than 3 million base pairs, region of copy neutral LOH 3p21.3. We analyzed the copy number of genes of this cluster in the study cohort and found that in B-ALL there are no duplications of chromosome fragments that include this cluster. LOH was found in only one patient, another patient had a 3p deletion, and five patients had monosomy of chromosome 3. Thus, loss of the TSG cluster 3p21.3 haplotype was observed in seven patients (19%).

### 2.7. Copy Number Alterations and cnLOH in Genes Involved in the Pathogenesis of B-ALL

Gene CNA in tumor cells may have clinical significance and influence survival. Therefore, we selected a panel of genes including *IKZF1*, *CDKN2A*/*B*, *PAX5*, *BTG1*, *TBLXR1*, *RAG1*, *RAG2* and *ETV6* that were described as being involved in the pathogenesis of B-ALL previously, we expanded the panel by adding genes that were found to have minor aberrations in patients of this cohort, and we analyzed gene copy number alterations in patients. The extended gene panel consists of *CDKN2A*/*B*, *DMRTA*, *DOCK8*, *TP53*, *SMARCA2*, *PAX5*, *XPA*, *FOXE1*, *HEMGN*, *USP45*, *RUNX1*, *NF1*, *IGF2BP1*, *ERG*, *TMPRSS2*, *CRLF2*, *FGFR3*, *FLNB*, *IKZF1*, *RUNX2*, *ARID1B*, *CIP2A*, *PIK3CA*, *ATM*, *RB1*, *BIRC3*, *MYC*, *IKZF3*, *ETV6*, *ZNF384*, *PTPRJ*, *CCL20*, *PAX3*, *MTCH2*, *TCF3*, *IKZF2*, *BTG1*, *BTG2*, *RAG1*, *RAG2*, *ELK3*, *SH2B3*, *EP300*, *MAP2K2*, *EBI3*, *MEF2D*, *MEF2C*, *CEBPA*, and *TBLXR1* genes.

For each gene, the role of the factor it encodes was analyzed using a literature search (in particular, the Pubmed Gene, GeneCards databases) and the association with the pathogenesis of tumors was confirmed. Next, we ranked genes by the frequency of occurrence of aberrant copy number (also including cnLOH) (Table 4 and Table S3). As expected, the genes of the *CDKN2A* and *DMRTA1* tumor suppressor gene cluster (9p21.3) were most involved in aberrant events.

The frequency of occurrence of gene copy number aberrations may be associated with its chromosomal localization. For example, the *DOCK8* gene (9p24.3) is located on the shorter arm of chromosome nine, 9p24.3, the first gene being 0.2 Mb from the p-telomere. In 14 patients, this gene fell into aberrant regions, including from one gene to an entire chromosome. We observed one biallelic deletion, five deletions, four cnLOH, and four duplications (Table S4).

### 2.8. The Prognostic Significance of Various Genetic Abnormalities

Our task was to assess the prognostic significance of the association of various genetic abnormalities with the course of the disease. The target end point was overall survival from the end of the induction course (70 days). (This landmark point was chosen to exclude the impact of early failures of infectious lethality.) To solve this problem, we used three approaches. At the first stage, we conducted a univariate event analysis of the association of overall survival with the molecular karyotype for each gene from the selected panel, as well as with the presence of the following known prognostically unfavorable cytogenetic abnormalities: t(4;11)—as a most unfavorable event—and hyperdiploidy, t(1;19), and t(7;14)—as favorable events. According to the results of univariate analysis, along with the well-known unfavorable prognosis factor t(4;11), other aberrations were noted. Abnormalities in the BIRC3 (*p* = 0.06) and in the ATM (*p* = 0.06) may be prognostically significant, and it is possible that increased copy number (Gain) in the ATM and BIRC3 genes is associated with an unfavorable outcome of the disease. The results of the analysis are presented in Figure S1. Moreover, in our sample, these two abnormalities are combined, the group with the presence of ATM gain consisted of three patients, the group with the presence of BIRC3 gain consisted of three patients, and in these three patients ATM and BIRC3 gains were detected simultaneously. Interestingly, these same three patients had a duplication of the KMT2A gene (MLL), located at the same locus 11q22.3. Deletion of this gene was detected only in one patient N°7, also combined with deletion of BIRC3 and ATM. However, due to the low occurrence of KMT2A aberrations in the cohort (less than six events), copy number aberrations of this gene were not included in the statistical analysis.

At the second stage, we conducted a multivariate analysis of overall survival (Cox model) with stepwise feature selection. As candidates, we used the status of “allele loss” including also cnLOH (yes-no) and the status of “duplication/amplification” (yes-no) for each gene from the selected panel, as well as the presence of hyperdiploidy, the presence of t(4;11), t(1;19) and t(7;14) translocations. Two features were confidently selected into the resulting model: the presence of ATM gene gain (OR = 8.08 (95% CI 1.05–50.0) *p* = 0.0233) and the presence of t(4;11) translocation (OR = 5.97 (95% CI 0.76–38.46) *p* = 0.056).

**Figure 6:**
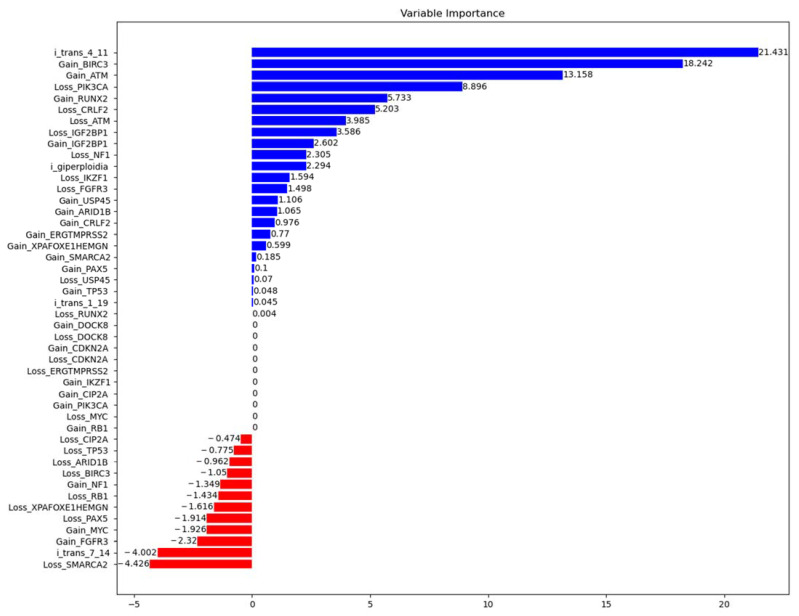
Random survival forests output—the variable importance (VIMP) values for gene and event signatures in prediction of overall survival for B-ALL patients (*n* = 36). The factors on it are ranked (from bottom to top) in increasing order of the variable importance. Red color represents favorable events with negative variable importance and blue color represents unfavorable events.

Also, as a separate analysis, we used the statistical method of Random Survival Forests to select the most significant prognostic features from a variety of features. Figure 6 presents the results of this analysis. The factors on it are ranked (from bottom to top) in increasing order of the possible influence on the overall survival prognosis. The most important in terms of possible influence according to the results of this analysis are the presence of the t(4;11) translocation and the presence of BIRC3 and ATM gene gains. As expected, one of the most favorable events was the t (7;14) translocation. Despite the fact that some factors have a high frequency of occurrence, they have zero variable importance and their impact on the prognosis was not detected (for example, loss of CDKN2A/B) according to the results of the analysis. Thus, all three analysis methods used showed fairly similar results. Understanding the limitations of the analysis due to the sample size and small number of genetic events, we can cautiously assume that BIRC3 and ATM genes gains are prognostically unfavorable in the B-ALL group. Taking into account the combination of BIRC3, ATM and KMT2A gene duplications, it can be assumed that duplications of other genes located in this aberrant region may also be associated with the most unfavorable prognostic events. To confirm this hypothesis, continued observation and expansion of the patient sample are required.

## 3. Discussion

Acute lymphoblastic leukemia (ALL) is a malignancy of immature lymphoid cells primarily associated with various chromosomal aberrations. Clinical standards for studying the genetic profile of a patient’s tumor cells include a combination of conventional karyotype and FISH analysis of the most common translocations. CMA is a high-throughput method for the whole-genome analysis of CNA that complements the diagnostic picture and is gradually being implemented into routine clinical practice [7].

In tumor pathogenesis, DNA copy number alterations can have a significant impact on gene expression and contribute to the development and progression of the disease, for example, deletion of tumor suppressor genes, amplification of oncogenes, and even drug resistance genes [8]. Shao et al. have shown close correlation between CNA and differential gene expression, revealing the qualitative relationship between genetic variation and its downstream effect, especially for oncogenes and tumor suppressor genes [9]. For this pan-cancer study, the authors used The Cancer Genome Atlas (TCGA) data for 31 cancer types from 9159 samples. In oncogenetics, CNA are divided into two classes based on their size: large-scale, also known as chromosome arm-level variants, covering >25%, and focal variants, defined as small regions of the genome, usually no more than 3 Mb in size, containing up to several genes [10]. Both types of CNA are important in the context of disease, but the relatively small size and low gene content make focal CNA more suitable for identifying candidate driver genes [11,12]. CNA analysis is an important aspect of molecular diagnostics in oncology. It has been shown that recurrent deletions are usually overrepresented in tumor suppressor genes and underrepresented in oncogenes [13]. Aberrations in gene copy number may indicate therapeutic targets or markers of drug resistance in some tumor types [14]. Despite the generally accepted rules for clinical assessment of the significance of CNA length, short somatic copy number changes cannot be ignored in research work—they may contain candidate genes, or the presence of a large number of these events may be an independent prognostic criterion [15].

Clonal heterogeneity of a tumor is a poor prognostic factor in ALL if three or more tumor clones are identified [16]; however, the CMA method, although it distinguishes the proportion of genomes with a certain aberration at a level of 15% (0.15), does not provide information about the combination of aberrations with different burdens in one or more tumor clones. Typically, clonal heterogeneity is analyzed based on variant allele frequency (VAF) determined in NGS and based on flow cytometry results in combination with clinical data.

Another detrimental factor, LOH 6p involving HLA locus is associated with a reduced ability of neoantigen presentation, thereby aiding tumor evasion from immune surveillance. Cn LOH 6p is frequently detected (20–40%) in a variety of tumors, and it is likely that this is a widespread mechanism that occurs regardless of tumor origin [17]. However, in the B-ALL cohort we studied, cnLOH 6p occurred in only one patient.

Many tumor suppressor genes (TSGs) are located in the small 3p21.3 genomic region. They may be involved, perhaps with varying roles, in different types of tumors [18,19,20,21]. However, in B-ALL studies, there are practically no references to aberrations involving this gene cluster [22].

Deletions of *CDKN2A*/*B* occur frequently in both childhood and adult ALL, with an incidence of 30–50%. The prognostic value of *CDKN2A*/*B* deletions has been widely investigated in numerous studies, but the results remain controversial [23]. Regarding the 9p21 cluster, Piskunova et al. investigated the association of 9p deletion with overall survival in patients with Ph-negative ALL treated according to RALL-2009 protocol. The prevalence of the *CDKN2A* deletion in the studied population was 24.3% (27 from 110 cases). Analysis of long-term treatment results showed that the presence of *CDKN2A*/*9p21* deletion did not affect prognosis and survival in adults with ALL [24].

The next occurrent copy number abberation in our cohort involves *DOCK8* gene that is crucial for the survival and function of various immune-related cells. However, the critical role of DOCK8 protein on tumorigenesis through regulating immunity is poorly understood. Accumulating evidence indicated that DOCK8 could affect tumorigenesis by regulating the immunity through immune cells, including NK cells, T cells, B cells and dendritic cells. Deletion or down-regulation of *DOCK8* was detected in leukemia, lung cancer, renal cell carcinoma, low-grade gliomas and childhood hairy cell astrocytoma. It has been shown that DOCK8 promotes the mesenchymal-type movement of hepatocellular carcinoma (HCC) cells, and the expression of *DOCK8* is negatively correlated with the occurrence of HCC [25]. In our patient cohort, *DOCK8* deletions and duplications were not associated with adverse events according to statistical analysis (Figure 6).

Our work focused on analyzing candidate genes with copy number alterations and assessing the clinical significance of these events. Duplications of two candidate genes, *BIRC3* and *ATM*, were identified as the most unfavorable prognostic events in the B-ALL group, comparable in significance to the t(4;11) translocation. BIRC3 is a multi-functional protein that regulates not only caspases and apoptosis, but also modulates inflammatory signaling and immunity, mitogenic kinase signaling and cell proliferation, as well as cell invasion and metastasis. BIRC3 acts as an E3 ubiquitin-protein ligase regulating both canonical and non-canonical NF-kappa-B signaling by acting in opposite directions. ATM is an important cell cycle checkpoint kinase. Thus, it functions as a regulator of a wide variety of downstream proteins, including tumor suppressor proteins p53 and BRCA1, checkpoint kinase CHK2, checkpoint proteins RAD17 and RAD9, and DNA repair protein NBS1. *BIRC3* and *ATM* genes are located in the same locus 11q22.2-3, so changes in their copy numbers are expected to be combined in most cases. Previously, a number of authors noted the deletion of the *BIRC3* gene in hematological malignancies, in particular in chronic lymphocytic leukemia (CLL), as an unfavorable prognostic marker. CLL patients harboring 11q22.3 deletion, are characterized by a rapid disease progression. One of the suggested genes to be involved in the pathogenesis of this deletion is the *BIRC3* gene, a negative regulator of NF-κB, which is monoallelically deleted in ~80% of del(11q) CLL cases. In addition, truncating mutations in the remaining allele of this gene can lead to BIRC3 biallelic inactivation, which accounts for marked reduced survival in CLL [26]. Evidently, the deletion or duplication of *BIRC3* may be observed rarely in B-ALL patients [27]. In our work, in addition to duplications, allelic loss was also noted, two patients had a deletion, and one had a cnLOH, but this event was not selected as the most significant by any of the statistical methods. Wu et al. identified amplification of 11q22.2 as prevailing copy-number alterations associated with strong overexpression of the *YAP1*, *BIRC2*, and *BIRC3* cancer-related genes in head-and-neck squamous cell carcinomas (HNSCCs) in patients with Fanconi anemia. Authors found the drug AZD5582, a known small molecule inhibitor of *BIRC2-3*, to selectively kill FA tumor cells that overexpressed BIRC2-3. Therefore, chemotherapeutic inhibition of overexpressed BIRC2-3 may provide the basis for an approach to develop a clinically realistic treatment of FA-HNSCCs that carry 11q22.2-3 amplifications [28]. Perhaps, in the future, this approach will be applicable to B-ALL with overexpression of certain factors.

Between the *BIRC3* and *ATM* genes there are about 30 more genes described in OMIM. We assume that duplication of any of these, when assessed using our chosen statistical algorithm, would be the most unfavorable event. Therefore, it is necessary to approach the selection of candidate genes with extreme caution, and perhaps adverse events should be determined not by genes, but by DNA loci and the nature of the event.

The results of microarray analysis in our work were obtained on a small group of patients. However, patients had a common diagnosis, received therapy according to one protocol and recruited in a limited time period. Therefore, it is a homogeneous sample suitable for correctly assessing the genetic events’ association with the outcome of therapy and for assessing risks. The introduction of reference events into the statistical analysis—translocations with known prognostic significance, favorable and unfavorable—and ranking them in a general series of signs according to the possible impact on overall survival adds credibility to our results. It seems to us that we were able to avoid errors in the statistical analysis [29], and the results obtained are consistent with the results of similar studies by our colleagues.

## 4. Materials and Methods

The study included 36 patients with Ph-negative B-ALL who received therapy at the National Medical Research Center for Hematology according to the RALL-2016m protocol from 2019 to 2023 and had available tumor DNA material at the onset of the disease. The RALL-2016 protocol is a modification of the previous RALL-2009/2016 protocols, based on the principle of low intensity and non-interruption treatment. MRD assessment is carried out on days +70 and +133 of therapy in accordance with the protocol. In case of MRD+, on day 70, the RALL-2016m protocol considered the application of the anti-CD-19 bispecific antibody blinatumomab. Patients included in the study provided informed consent for the use of their biomaterials in the research project. The work was approved by the local ethics committee.

All patients included in the protocol underwent immunophenotyping, cytogenetic and molecular tests of bone marrow samples at the onset of the disease. Bone marrow cells obtained from patients during the initial examination were analyzed using G-differential chromosome staining and FISH. The FISH method was used to detect the t (9;22) translocation (XL *BCR*/*ABL1* plus Translocation—Dual Fusion Probe (Metasystems, Altlussheim,

Germany), *BCR*/*ABL* Translocation, Dual Fusion Probe (Aquarius^®^ Cytocell, Cambridge, United Kingdom) and *KMT2A* (*MLL*) gene rearrangement (XL *MLL* plus Break Apart Probe (Metasystems, Altlussheim, Germany)). After a standard cytogenetic test, additional FISH tests were performed to identify rearrangements of the *IGH* (*IGH* Breakapart Probe (Aquarius^®^ Cytocell)), *cMYC* (*c-MYC* Breakapart Probe (Aquarius^®^ Cytocell)), *E2A* (XL *E2A* Break Apart Probe (Metasystems)), *TP53* (XL P53 Deletion Probe (Metasystems)), *ETV6* (ON *ETV6* (TEL) (12p13) Break (Poseidon™ Kreatech, Amsterdam, Netherlands)) and *CDKN2A* (XL *CDKN2A* Deletion Probe (Metasystems)) genes. The karyotype and results of FISH analysis were described in accordance with the criteria of the International Cytogenomic Nomenclature ISCN, 2020 [30].

In patients who met B-ALL criteria, we performed an additional analysis to determine the immunophenotype associated with leukemia for further assessment of MRD using flow cytometry. MRD was assessed using the “different from normal” method, which is based on the knowledge of normal hematopoietic cell immunophenotype [31]. Tests before 2020 were performed with BD FACSCanto II flow cytometer with 2-tube 6-color panel, which includes antibodies against CD19, CD45, CD38, CD10, CD34, CD58 and CD20 (all produced by BD Biosciences, USA, except CD58 (Beckman Coulter)). Studies after 2021 were performed with BC CytoFLEX flow cytometer with single-tube 9-color panel, which includes antibodies against CD19, CD45, CD38, CD10, CD34, CD58, CD20, CD22 and CD24 (all produced by Biolegend, USA, except CD10 (BD Biosciences, San Jose, CA 95131, USA) and CD58 (Beckman Coulter, Brea, CA 92821, United States).

The cases with leukemic cells comprising distinct immunophenotype homogenous populations with different expression of at least two antigens (for example, CD34+CD10− and CD34−CD10+ subpopulations) were marked as cases with multiple leukemic clones.

MRD was assessed at the end of induction (day 70) using 6- or 10-color flow cytometry of the bone marrow specimens.

CMA was carried out with Thermo Fisher Scientific (Santa Clara, CA 95151, USA) equipment using the CytoScan™ HT-CMA 96F array SNP-oligonucleotide microarray (Thermo Fisher Scientific, USA) in accordance with the manufacturer’s protocol. The analysis was performed at the “Genomed” laboratory of Molecular Pathology (Moscow, Russia). Material for analysis—DNA isolated from bone marrow cells in patients with ALL before therapy, in an amount of not less than 100 ng and not more than 200 ng with an A260/A280 ratio of not less than 1.8 and reference male DNA of a similar concentration (Thermo Fisher Scientific, USA). The scanning results were processed with the Multi Sample Viewer Software (v.1.1.0.11) and Chromosome Analysis Suite (ChAS 4.3.0.71) (Thermo Fisher Scientific, USA). Cutoff of ≥5 Mb for a CNA size was used according to Schoumans et al. [4]. CNAs with a distance ≤5 Mb between each other were counted as one event.

The achievement of MRD-negative status on day 70 (end of induction) and overall and disease-free survival from the end of induction (day 70) were used as end points to study the prognostic significance of various aberrations. This choice of the starting point was thought to neutralize the influence of early mortality during induction courses associated with infectious complications. For overall survival analysis, the time interval was measured from the end of induction to the date of death or last contact. For the analysis of disease-free survival, the time interval for patients who achieved remission on induction courses was measured from the end of induction to the date of the first adverse event (relapse, death) or the date of last contact.

Analysis of overall survival and disease-free survival was performed using Kaplan–Meier estimates and the Log-Rank test was used to compare estimates between groups. For multivariate analysis, we used the proportional hazards model (Cox model). The hypotheses about differences in the distributions of categorical features in comparison groups were tested using contingency tables. To assess the significance of frequency differences, Fisher’s exact test was used. Machine learning method, random forest, has shown good performance in oncology applications, especially in case of large dimension of variable space and moderate patient sample size [32,33]. We also used the Random Survival Forests method as a separate analysis method for ordering and selecting the most significant prognostic features from the set of features. All calculations were made using SAS 9.4 and R 4.2.3. Random Survival Forests method was performed using R package “random Forest SRC” [34].

## 5. Conclusions

We did not identify significant associations between the frequency of achieving MRD-negative status and genetic aberrations. We also did not reveal a significant association of OS with tumor clonal heterogeneity at the disease onset. *BIRC3* and *ATM* gene gains are prognostically unfavorable in the B-ALL group. Taking into account the combination of *BIRC3*, *ATM* and *KMT2A* gene duplications, it can be assumed that duplications of other genes located in this aberrant region 11q22.2-3 may also be associated with the most unfavorable prognostic events. It makes sense to assess the association between gene deletions and duplications and clinical outcome, taking into account the chromosomal localization of genes and the involvement of neighboring genes in the loci of aberrations. Focusing on microdeletions and microduplications reproduced in the tumor genome in different patients allows us to identify new candidate genes that drive tumorigenesis.

## Figures and Tables

**Table 1:** The main characteristics of the patients.

Parameter	B-ALL (*n* = 36)
Male:Female	21:15
Age, median	34.1 (19–53) years
Leukocytes, 10 × 9/L	7.66 (1.09–466.53)
LDH	624 (148–7348.8)
Blast cells in peripheral blood, %	40 (0–95)
Blast cells in the bone marrow, %	88.8 (29–98)
Immunophenotype, EGIL, WHO	B-I 5 (13.8%)
	B-II 30 (83.3%)
	B-III 1 (2.7%)
	B-IV 0 (0%)
Standard cytogenetics	36
+ mitosis	35 (97.2%)
− mitosis	1 (2.8%)
Karyotype	35
Normal	7 (20.0%)
Abnormal:	28 (80.0%)
CNS leukemia	3 (8.3%)
Extramedullary disease	14 (38.8%)
MRD-status (+70 day)	34
MRD+	18
MRD-	16
CR:	
After 2nd induction (+70 day)	16
Refractory disease	1
Early Death	1

LDH, lactate dehydrogenase; EGIL, European Group on Immunological Classification of Leukemia; WHO, World Health Organization; CNS, central nervous system; CR, complete remission.

**Table 2:** Cytogenetic and molecular karyotype of patients.

N°	Sex	Age	Diagnosis	CCA Data [mitoses count]	CMA Data	FISH
1	f	31	B-II	no mitosis	(X,1-22)×2	n/rvl t(9;22)(q34;q11); t(11q23)/*MLL*
2	f	26	B-II	46,XX,t(4;11)(q21;q23)[7]/46,XX [13]	(X,1-22)×2	21% t(11q23)/*MLL*
						n/rvl t(9;22)(q34;q11)
3	f	54	B-II	50,XX,+X,+8,+9,i(9)(q10),+22[10]/46,XX [4]	(X)×3, 2p16.3×1, 5q35.2q35.3×2 hmz, 7p12.3p12.1×1, (8)×3, 9p24.3p21.3×2 hmz, 9p21.3×0, 9p21.3p13.1×2 hmz, (9q)×4, 17q11.2q11.2×2 hmz, (22) ×3	94%++ABL/9q34 ++*BCR*/22q11
						n/rvlt(9;22)(q34;q11); t(11q23)/*MLL*
4	f	34	B-I	47,XX,+X,t(4;11)(q21;q23)[17]/46,XX [3]	(X)x3[0.6],1p21.2p21.1x2 hmz,3q25.31q26.1x2 hmz, 3p21.1p14.2×2 hmz,3p22.1p21.31×2 hmz, 4q34.3q34.3×2 hmz,5p13.1q11.2×2 hmz, 6p22.3p22.3×2 hmz,8p23.1p22)×2 hmz, 9q22.31q31.1×2 hmz,10q25.3q26.13×2 hmz, 10q25.1q25.2×2 hmz,11q22.3q23.2×2 hmz	90% t(11q23)/*MLL*
						n/rvl t(9;22)(q34;q11)
5	f	34	B-II	46,XX [20]	(X)×1 [0.2], 2q24.1q24.1×2 hmz, (3)×1 [0.19], 4q31.3q32.1×1 [0.2], (7)×1 [0.19],7q31.2q31.31×2 hmz, (8)×3 [0.31], (9)×1 [0.19], (10)×3 [0.29],	12% + *MLL*/11q23, +*BCR*/22q11
					(14)×3 [0.3], (15)×1 [0.2],(16)x1 [0.19],(20)×1 [0.17],(21)x3 [0.31]	n/rvl t(9;22)(q34;q11); t(11q23)/*MLL*
6	m	32	B-II	46,XY,der(6),-11,+mar or der(11?)[6]/46,XY [14]	3p21.31×1,6q16.1q22.1×1, 9p24.3×3,11p14.3p12×1, 11p11.2q12.1×1,11q14.2q14.3x1, 11q22.1q23.3×1,11q23.3q24.1×1, 11q24.1×1,12p13.31×3, 19p13.3×1	n/rvl t(9;22)(q34;q11); t(11q23)/*MLL*
7	f	24	B-II	81-85,XX, ?-X, del (6)(q22)*2, add(11)(q2?5),+3-4 mar, inc [cp2]/	Xp22.33p11.3×1 [0.2],Xp11.3q28×1 [0.5], 3p26.3p12.2×1 [0.3], 3p12.1p11.1×2 hmz,	n/rvl t(9;22)(q34;q11); t(11q23)/*MLL*
				46,XX [18]	3q25.33q29×3 [0.3], 6q16.3q24.3×1 [0.6], (9)×1 [0.21], (11)×1 [0.3], 12p13.33p13.2×1 [0.22], 12p13.2p12.1×2 hmz [0.5], 12p11.23q22×3 [0.3], 12q22q24.33×1 [0.2], (15)×1 [0.72], 17p13.3q21.31×1 [0.2], 17q21.31q25.3×2 hmz [0.5], 21q22.12q22.3×3 [0.3]	
8	m	41	B-I	46,XY,t(4;11)(q21;q23)[3]	1p31.1p31.1×2hmz, 5q23.1q23.1x2 hmz	98% t(4;11)(q21;q23)
						n/rvl t(9;22)(q34;q11)
9	f	29	B-II	56,XX,+X,+4,+4,+6,+10,+14,+17,+18,+21,	(X)×3, (4)×5, (6)×3, (10)×3,	n/rvl t(9;22)(q34;q11); t(11q23)/*MLL*
				+21[18]/46,XX [2]	11p11.2p11.12×2 hmz, (14) ×3, (17) ×3, (18) ×3, (21) ×5	
10	m	53	B-I	46,XY,t(4;11)(q21;q23) [20]	2q36.3q37.1×2 hmz, 4q12q13.2×2 hmz,	88% t(11q23)/*MLL*
					9p24.3p13.3×2 hmz, 9p21.3×1	
12	f	36	B-II	46,XX,add(14)(q32) or t(14;?)(q32;?)	14q31.2q32.33×3	92% t/del14q32/IGH(telomeric part);
						n/rvl t(9;22)(q34;q11); t(11q23)/*MLL* t(12p13)/*ETV6*
13	m	20	BIII/BIV	52~54,XY,+X,+4,+8,+14,+14,+18,+21,	(X) ×2 [0.83], (4) ×3, 6q15q23.1×1[0.36], (8) ×3, 9p21.3×0, 9p21.3p21.2×1, 9q22.33q31.1×1, 11q22.3×1, 13q14.2q31.1×1, (14) ×3, (18)x3, (21) ×3	94% +*cMYC*/8q24
				+mar[cp16]/46,XY [4]		n/rvl t(8q24)/*cMYC*; t(11q23)/*MLL*; t(9;22)(q34;q11)
14	m	22	B-II	56,XY,+X,+Y,+4,+6,+8,+10,+14,+17,+18,	(X,Y)x1,(1-22)x2	n/rvl t(9;22)(q34;q11); t(11q23)/*MLL*
				+21[9]/46,XY [11]		
15	m	19	B-II	54~56<2n>,XY,+X,+4,+6,+9,+10,+14,+15,+17,+21,+21[cp13]/	(X)×2 [0.81], 3p21.31p21.1×2 hmz, (4) ×3 [0.72], (6)×3 [0.69], (9)×3 [0.42], (10)×3, (14)×3, (17)×3, (18)×3, 19p13.3p13.3×1 [0.4],	30% +*ABL*/9q34
				54~56,idem,add(19)(p?q?)[5]/46,XY [2]	19p13.2p13.12×3 [0.3], (21) ×5	n/rvl t(9;22)(q34;q11) t(11q23)/*MLL*
16	m	20	B-II	52~54,XY,+X,+4,+6,+?10,+17,+18,+20,+21,+mar[cp5]/ 52~54, XY, +X, dup(1)(q3?1q44),+4,+6,+?10, +17, +18, +20, +21[cp3]/46,XY [5 ]	(X)×2 [0.78], (4)×3, (6)×3, (10)×3, (17)×3, (18)×5,(21)×3	n/rvl t(9;22)(q34;q11); t(11q23)/*MLL*
17	m	22	B-II	46,XY [20]	(2)×1, (3)×1, (4)×1, (7)×1, (9)×1, (12)×1, (13)×1, (16)×1, (17)×1, (20)×1	n/d
18	f	28	B-II	46,XX,der(19)t(1;19)(q23;p13), inc[cp15]/	1q21.1q23.3×3 [0.7], 1q23.3q43×5 [0.5], 1q43q44×4 [0.6],	28% t/del (19p13)/*E2A* (telomeric part)
				46,XX [5]	9p24.3p12x1 [0.31], 19p13.3x1	
19	m	44	B-II	36,X,-Y,-2,-3,der(4),-7,der(12),-13,-15,-16,-17, -20x2,-22, +mar[cp6]/46,XY [14]	(Y) ×0 [0.8], (2) ×1, (3) ×1, 4q26q26×2 hmz, 5p13.2p13.1×1, (7)x1, 11q13.3q25×3, 12p13.33p11.22×1, 13q12.11q22.3×1, 13q22.3q34×1 [0.2],	96% del (22q11)/*BCR*
					(15) ×1,(16) ×1,(17) ×1,18q11.2×3,(20) ×1,(22) ×1	n/rvl t(9;22)(q34;q11)
21	f	46	B-II	46,X,-X,	(X) ×1 [0.1], 1p36.13p34.31×1 [0.2],1q23.3q44×3, 3q13.12q13.2×1 [0.5],4q21.22q28.1×1 [0.2], 4q35.1q35.2×1 [0.2],5q11.2q12.1×1 [0.2], 6q11.1q21×1 [0.5],6q22.32q22.33×2 hmz, 6q24.3q25.1×1 [0.5],6q25.3q27×1 [0.5], 8q11.1q11.21×2 hmz,9p24.3p24.2×0 [0.3],9p24.1p21.3×1, 9p21.3p21.3×0 [0.3], 9p21.3p11.2×1,9q21.11q34.3×3, 13q12.11q12.12×1 [0.5],13q13.3q31.1×1 [0.5], 18p11.32p11.31×1 [0.5],19p13.3×1,(21) ×3	n/d
				add(1)(p3?3),-4,		
				i(9)(q10),		
				der(19)t(1;19)(q23;p13),+?21,+mar [15]/		
				46,XX [5]		
22	f	44	B-II	46,XX[29]/	7q35q36.2×1 [0.3],9p21.3p21.2×1 [0.4]	n/rvl t(9;22)(q34;q11)
				47,XX,+12[1]		
23	f	39	B-II	52~54,?XX,+?6,+?11,+?17,+?18,+?20,+?21,	(X) ×3,(4) ×3,(6) ×3,(8) ×3, 9p24.3p13.1×2 hmz,9p21.3p21.3×0 [0.5],(10) ×3,(14) ×3,	n/rvl t(9;22)(q34;q11)
				inc[cp3]/46,XX [12]	14q23.1q23.2×2 hmz,(17) ×3,(18) ×3,(21) ×5	
24	f	36	B-II	44-45, X, -X,der(2),der(3),der(5),der(7),der(17),add(p11),+mar[cp5]/	Xp22.33p22.32×1 [0.37], 2p13.1p12×1 [0.31], 3p24.1p22.3×1 [0.27], 3q13.12q13.13×1 [0.42], 5q11.2q12.1×1 [0.26], 5q13.3q14.1×1 [0.31], 7p12.2p12.1×1 [0.44], 13q14.2q14.3×1 [0.2], 17p13.3p11.1×1 [0.35]	n/rvl t(11q23)/*MLL*, t(9;22)(q34;q11
				46,XX [15]		
25	m	26	B-II	45,XY,-7,der(14) t(7;14)(q11;q?32), del(17)(p10)[6]/	2p11.2p11.2×1, 7p14.1p11.2×1 [0.16], 7q34×0, 9p24.3p13.2×2 hmz, 9p21.3×0,16p13.3×1, 17p13.3p11.2×1	90% del (17p13)/*TP53*;
				46,XY [14]		n/rvl t(9;22)(q34;q11); t(14q32)/IgH; t(11q23)/*MLL*
26	f	38	B-II	46,XY [20]	5 q14.3q14.3×2 hmz	n/rvl t(11q23)/*MLL*; t(9;22)(q34;q11)
27	m	23	B-I	46,XY,der(19)[20]	(X,Y) ×1,(1-22) ×2	n/rvl t(9;22)(q34;q11); t(11q23)/*MLL*; t(19p13)/ *E2A*
28	f	23	B-II	46,XX,add(1)(q44),der(9), inc [9]/46,XX [11]	1q21.1q32.1×3, 6q16.2q16.3×2 hmz, 9q21.13q31.1×1, 11q22.1q22.3×2 hmz	n/rvl t(9;22)(q34;q11)
29	m	42	B-II	46,XY [13]	11p11.2p11.12×2 hmz, 21q21.1q21.3×2 hmz	n/rvl t(11q23)/*MLL*; t(9;22)(q34;q11)
30	m	21	B-II	57,XY,+X,+4,+6,+8,+10,+14,+17,+18,+21x2,+mar[14]	(X)×2 [0.81],(Y)×0 [0.13],(4)×3, (6)×3,(8)×3,(9)×3 [0.19],(10)×3,(14)×3,(17) ×3,(18) ×3,(21)×4	20% +*ABL*/9q34
						n/rvl t(9;22)(q34;q11) t(11q23)/*MLL*
31	f	47	B-II	46,XX,del(9)(p?21)[5]/46,XX[15]	2q36.1×1,3q26.32q26.32×0, 3q26.32q26.33×1	11% del(9p21)/ *CDKN2A*
						n/rvl t(9;22)(q34;q11)
32	f	24	B-II	46, XX [20]	(1q)×3 [0.19],(2)×1 [0.16],(3)×1 [0.15],(7)×1 [0.15],(8)×3 [0.15], 9p24.3q22.32×3 [0.12],9q22.32q34.3×1 [0.17],(10)×3 [0.43] (11)×3 [0.15],(12) ×1 [0.18],(13)×1 [0.15],(14)×3 [0.2],(15)×1 [0.18],(16)×1 [0.15], (18p)×3 [0.46],(18q)×3 [0.25],19p13.3p13.11×3 [0.15], 19p13.11q13.43×1 [0.2],(21)×3 [0.19]	5% +*MLL*/11q23
						n/rvl t(9;22)(q34;q11) t(11q23)/*MLL*
34	f	44	B-II	46,XY,der(9),add(13)(p11),add(15)(p11),	2q32.3q33.1×2 hmz,9p24.3×3,(21)×3 [0.15]	n/rvl t(9;22)(q34;q11); t(11q23)/*MLL*
				der(17) or mar,der(22) or mar,inc[9]/46,XY[2]		
35	f	40	B-II	46,XX [20]	(X,1-22)×2	n/rvl t(9;22)(q34;q11); t(11q23)/*MLL*;
36	f	30	B-II	?54-55,XX,?+4,+8,?+8 or 10,+9,+11,+?14,+21,+21,+mar[cp6]/46,XX[19].	(X,1-22)×2	90%+ *MLL* (11q23), +*ABL* (9q34)
						n/rvl t(11q23)/*MLL*; t(9;22)(q34;q11)
37	m	27	B-II	46,der(X),Y,del(6)(q22), ?der(16)[20]	6p21.1×1, 6q14.3q22.31×1,6q25.2q25.3×1, 12p13.2p13.1×1,19q12×1, 19q13.11×1	n/rvl t(9;22)(q34;q11); t(11q23)/*MLL*
38	f	38	B-II	46,XX[20]	2p11.2×1, 3p14.3×0, 12q13.12×1, 21q21.1×1	n/rvl t(9;22)(q34;q11)
39	m	41	B-I	34~38,XY,	(3) ×1 [0.5], (4) ×1 [0.5], (5) ×1 [0.5],6p22.2p21.32×2 hmz,(7) ×1 [0.5],(9) ×1 [0.5], 11q14.1q25×3 [0.6],(15) ×1 [0.5],(16) ×1 [0.5],(17) ×1 [0.5],(20) ×1 [0.5]	n/d
				+2-3mar[12]/46,XY[8]		

* n/rvl not revealed, n/d no data; +one additional signal of gene, ++two additional signals of locus. Patient numbering in a cohort is not continuous but individual. There are 36 patients in the cohort. Patients 11, 20, and 33 were excluded from the study due to other leukemia or pretreatment. The values of the proportion of additional clones determined by FISH, as well as the proportion of aberrations that differ significantly from the rest identified in the patient by CMA, are highlighted in red.

**Table 3:** Genes lost in the tumor genome as a result of biallelic/homozygous deletions in patients with B-ALL.

N° Pat	Locus	Genes Affected by Biallelic/Homozygous Deletion	Nature of Loss
38	3p14.3	*FLNB*	biallelic
31	3q26.32	*TBL1XR1*, *KCNMB2*	biallelic
25	7q34	*TCAF2*, *PRSS1*, *PRSS2*	biallelic
25	9p21.3	*MTAP*, *CDKN2A*, *CDKN2B-AS1*, *CDKN2B*, *DMRTA1*	homozygous
13	9p21.3	*IFNB1*, *IFNW1*, *IFNA21*, *IFNA4*, *IFNA7*, *IFNA10*, *IFNA16*, *IFNA17*, *IFNA14*, *IFNA5*, *KLHL9*, *IFNA6*, *IFNA13*, *IFNA2*, *IFNA8*, *IFNA1*, *MIR31HG*, *IFNE*, *MIR31*, *MTAP*, *CDKN2A*, *CDKN2B-AS1*, *CDKN2B*, *DMRTA1*, *ELAVL2*, *IZUMO3*	biallelic
23	9p21.3	*MTAP*, *CDKN2A*, *CDKN2B-AS1*, *CDKN2B*, *DMRTA1*	homozygous
3	9p21.3	*CDKN2A* , *CDKN2B-AS1*, *CDKN2B*, *DMRTA1*, *ELAVL2*	homozygous
21	9p21.3	*DMRTA1*	biallelic
21	9p24.3	*DOCK8*, *KANK1*, *DMRT1*, *DMRT3*, *DMRT2*, *SMARCA2*, *VLDLR*, *KCNV2*, *PUM3*, *RFX3*, *GLIS3*, *SLC1A1*	biallelic

9p21.3 tumor suppressor cluster genes are highlighted in red.

**Table 4:** Genes that most often fall into areas of DNA copy number changes, their localization and type of events (deletion, cnLOH, duplication).

Gene ^1^	Chr. Location	Count of Event	Type of Events
*CDKN2A*/*B*	9p21. 3	15	4loss0/8loss/3gain
*DMRTA*	9p21.3	15	4loss0/6loss/2cnLOH/3gain
*DOCK8*	9p24.3	15	1loss0/5loss/4cnLOH/5gain
*TP53*	17p13.1	13	7loss/6gain
*SMARCA2*	9p24.3	13	6loss/4cnLOH/3gain
*PAX5*	9p13	12	6loss/ 3cnLOH/ 3gain
*XPA*; *FOXE1*; *HEMGN*	9q22.33	12	7loss/1cnLOH/4gain
*USP45*	6q16.2	12	4loss/1cnLOH/7gain
*RUNX1*	21q22.2	11	7 gain/1gainX4/3gainX5
*NF1*	17q11.2	11	5loss/ 1cnLOH/ 5gain
*IGF2BP1*	17q21.32	10	4loss/1cnLOH/5gain
*ERG; TMPRSS2*	21q22.2	9	5gain/1gain4x/3gain5x
*CRLF2*	Xp22.23	9	4loss/ 5gain
*FGFR3*	4p16.3	9	2loss/7gain
*FLNB*	3p14.3	8	7 loss/1 cnLOH
*IKZF1*	7p12.2	8	8 loss
*RUNX2*	6p21.1	8	1loss/7gain
*ARID1B*	6q25.3	8	1loss/7gain
*CIP2A*	3q13.13	7	7 loss
*PIK3CA*	3q26.32	7	6loss/1gain
*ATM*	11q22.3	6	3loss/3gain
*RB1*	13q14.2	6	6 loss
*BIRC3*	11q22.3	6	2loss/1cnLOH/3gain
*MYC*	8q24.21	6	6gain

^1^ Genes for which 6 or more events were identified in the studied cohort are presented.

## Data Availability

Data are contained within the article and supplementary materials.

## Supplementary Materials

The supporting information can be downloaded at: https://www.mdpi.com/article/10.3390/ijms242417602/s1.

## References

[1] Parovichnikova E.N., Aleshina O.A., Troitskaya V.V., Chabaeva Y.A., Sokolov A.N., Isinova G.A., Kotova E.S., Akhmerzaeva Z.H., Klyasova G.A., Galtseva I.V. (2022). Comparison of the treatment results in adult patients with acute Ph-negative lymphoblastic leukemia on protocols of the Russian multicenter studies ALL-2009 and ALL-2016. Russ. J. Hematol. Transfusiology.

[2] Thiagalingam S., Foy R.L., Cheng K., Lee H.J., Thiagalingam A., Ponte J.F. (2002). Loss of heterozygosity as a predictor to map tumor suppressor genes in cancer: Molecular basis of its occurrence. Curr. Opin. Oncol..

[3] Makishima H., Maciejewski J.P. (2011). Pathogenesis and consequences of uniparental disomy in cancer. Clin. Cancer Res..

[4] Schoumans J., Suela J., Hastings R., Muehlematter D., Rack K., van den Berg E., Berna Beverloo H., Stevens-Kroef M. (2016). Guidelines for genomic array analysis in acquired haematological neoplastic disorders. Genes Chromosomes Cancer.

[5] Bandaru S., Zhou A.X., Zhang Y., Bergo M.O., Cao Y., Akyürek L.M. (2014). Targeting filamin B induces tumor growth and metastasis via enhanced activity of matrix metalloproteinase-9 and secretion of VEGF-A. Oncogenesis.

[6] Ma H.R., Cao L., Wang F., Cheng C., Jiang R., Zhou H., Xie Z., Wuermanbieke S., Qian Z. (2020). Filamin B extensively regulates transcription and alternative splicing, and is associated with apoptosis in HeLa cells. Oncol Rep..

[7] Mitrakos A., Kattamis A., Katsibardi K., Papadhimitriou S., Kitsiou-Tzeli S., Kanavakis E., Tzetis M. (2019). High resolution Chromosomal Microarray Analysis (CMA) enhances the genetic profile of pediatric B-cell Acute Lymphoblastic Leukemia patients. Leuk Res..

[8] Pös O., Radvanszky J., Buglyó G., Pös Z., Rusnakova D., Nagy B., Szemes T. (2021). DNA copy number variation: Main characteristics, evolutionary significance, and pathological aspects. Biomed J..

[9] Shao X., Lv N., Liao J., Long J., Xue R., Ai N., Xu D., Fan X. (2019). Copy number variation is highly correlated with differential gene expression: A pan-cancer study. BMC Med. Genet..

[10] Leary R.J., Lin J.C., Cummins J., Boca S., Wood L.D., Parsons D.W., Jones S., Sjöblom T., Park B.H., Parsons R. (2008). Integrated analysis of homozygous deletions, focal amplifications, and sequence alterations in breast and colorectal cancers. Proc. Natl. Acad. Sci. USA.

[11] Brosens R.P.M., Haan J.C., Carvalho B., Rustenburg F., Grabsch H., Quirke P., Engel A.F., Cuesta M.A., Maughan N., Flens M. (2010). Candidate driver genes in focal chromosomal aberrations of stage II colon cancer. J. Pathol..

[12] Krijgsman O., Carvalho B., Meijer G.A., Steenbergen R.D.M., Ylstra B. (2014). Focal chromosomal copy number aberrations in cancer-Needles in a genome haystack. Biochim. Biophys. Acta.

[13] Zhang L., Yuan Y., Lu K.H., Zhang L. (2016). Identification of recurrent focal copy number variations and their putative targeted driver genes in ovarian cancer. BMC Bioinf..

[14] Peng H., Lu L., Zhou Z., Liu J., Zhang D., Nan K., Zhao X., Li F., Tian L., Dong H. (2019). CNV detection from circulating tumor DNA in late stage non-small cell lung cancer patients. Genes.

[15] Murakami F., Tsuboi Y., Takahashi Y., Horimoto Y., Mogushi K., Ito T., Emi M., Matsubara D., Shibata T., Saito M. (2021). Short somatic alterations at the site of copy number variation in breast cancer. Cancer Sci..

[16] Lyu X.D., Guo Z., Li Y.W., Hu J.Y., Fan R.H., Song Y.P. (2020). Clonal heterogeneity and its prognostic significance in acute lymphoblastic leukemia. Zhonghua Nei Ke Za Zhi.

[17] Garrido M.A., Perea F., Vilchez J.R., Rodríguez T., Anderson P., Garrido F., Ruiz-Cabello F., Aptsiauri N. (2021). Copy Neutral LOH Affecting the Entire Chromosome 6 Is a Frequent Mechanism of HLA Class I Alterations in Cancer. Cancers.

[18] Ji L., Minna J.D., Roth J.A. (2005). 3p21.3 tumor suppressor cluster: Prospects for translational applications. Future Oncol..

[19] Oh J.J., West A.R., Fishbein M.C., Slamon D.J. (2002). A candidate tumor suppressor gene, H37, from the human lung cancer tumor suppressor locus 3p21.3. Cancer Res..

[20] Zhao L., Li R., Shao C., Li P., Liu J., Wang K. (2012). 3p21.3 tumor suppressor gene RBM5 inhibits growth of human prostate cancer PC-3 cells through apoptosis. World J. Surg. Oncol..

[21] Oh J.J., Taschereau E.O., Koegel A.K., Ginther C.L., Rotow J.K., Isfahani K.Z., Slamon D.J. (2010). RBM5/H37 tumor suppressor, located at the lung cancer hot spot 3p21.3, alters expression of genes involved in metastasis. Lung Cancer.

[22] Sinclair P.B., Blair H.H., Ryan S.L., Buechler L., Cheng J., Clayton J., Hanna R., Hollern S., Hawking Z., Bashton M. (2017). Dynamic clonal progression in xenografts of acute lymphoblastic leukemia with intrachromosomal amplification of chromosome 21. Haematologica.

[23] Zhang W., Kuang P., Liu T. (2019). Prognostic significance of CDKN2A/B deletions in acute lymphoblastic leukaemia: A meta-analysis. Ann Med..

[24] Piskunova I.S., Obukhova T.N., Parovichnikova E.N., Kulikov S.M., Gavrilina O.A., Lukyanova I.A., Savchenko V.G. (2017). CDKN2A/p16INK4a deletion is not a poor prognostic factor in adult acute lymphoblastic leukemia patiets treated according to protocol RALL-2009. Oncohematology.

[25] Zhang L., Cao Y., Dai X., Zhang X. (2022). Deciphering the role of DOCK8 in tumorigenesis by regulating immunity and the application of nanotechnology in DOCK8 deficiency therapy. Front. Pharmacol..

[26] Quijada-Álamo M., Hernández-Sánchez M., Rodríguez-Vicente A.E., Pérez-Carretero C., Rodríguez-Sánchez A., Martín-Izquierdo M., Alonso-Pérez V., García-Tuñón I., Bastida J.M., Vidal-Manceñido M.J. (2021). Biological significance of monoallelic and biallelic BIRC3 loss in del(11q) chronic lymphocytic leukemia progression. Blood Cancer J..

[27] Alhourani E., Othman M.A., Melo J.B., Carreira I.M., Grygalewicz B., Vujić D., Zecević Z., Joksić G., Glaser A., Pohle B. (2016). BIRC3 alterations in chronic and B-cell acute lymphocytic leukemia patients. Oncol. Lett..

[28] Wu Q., Berglund A.E., MacAulay R.J., Etame A.B. (2021). A Novel Role of BIRC3 in Stemness Reprogramming of Glioblastoma. Int. J. Mol. Sci..

[29] Dupuy A., Simon R.M. (2007). Critical review of published microarray studies for cancer outcome and guidelines on statistical analysis and reporting. J. Natl. Cancer Inst..

[30] McGowan-Jordan J., Ros J. (2020). ISCN 2020: An International System for Human Cytogenomic Nomenclature.

[31] Borowitz M.J., Wood B.L., Keeney M., Hedley B.D. (2022). Measurable Residual Disease Detection in B-Acute Lymphoblastic Leukemia: The Children’s Oncology Group (COG) Method. Curr. Protoc..

[32] Pickett K.L., Suresh K., Campbell K.R., Davis S., Juarez-Colunga E. (2021). Random survival forests for dynamic predictions of a time-to-event outcome using a longitudinal biomarker. BMC Med. Res. Methodol..

[33] Bohannan Z.S., Coffman F., Mitrofanova A. (2022). Random survival forest model identifies novel biomarkers of event-free survival in high-risk pediatric acute lymphoblastic leukemia. Comput. Struct Biotechnol. J..

[34] Ishwaran H., Kogalur U. (2023). Fast Unified Random Forests for Survival, Regression, and Classification (RF-SRC). R Package Version 3.2.2. https://cran.r-project.org/package=randomForestSRC.

