# Supplementary material for: DNA Copy Number Alterations and Copy Neutral Loss of Heterozygosity in Adult Ph-Negative Acute B-Lymphoblastic Leukemia: Focus on the Genes Involved
Source: Int J Mol Sci. 2023 Dec 18;24(24):17602. doi: 10.3390/ijms242417602 (PMC10744257; doi:10.3390/ijms242417602)
Supplement: Supplementary file 1 [file ijms-24-17602-s001.zip › Figure S1.pdf]

Gene	Frequency	Survival Estimates at 12 month (95% CI)	Hazard Ratio (95% CI)	p
**CDKN2A/B**				0.65
Norm	11 (31,4%)	0.84 (0.70-1.00)		
Gain	3 (8,6%)	1.00 (1.00-1.00)	Not estimated	
LOSS/LOH	21 (60%)	0.76 (0.51-1.00)	1.04 (0.19-5.59)	
**DOCK8***				0.53
Norm	21 (60%)	0.85 (0.71-1.00)		
Gain	5 (14,3%)	1.00 (1.00-1.00)	Not estimated	
LOSS/LOH	9 (25,7%)	0.71 (0.43-1.00)	1.28 (0.24-6.79)	
**TP53**				0.55
Norm	22 (62.8%)	0.85 (0.70-1.00)		
Gain	6 (17.1%)	1.00 (1.00-1.00)	Not estimated	
LOSS/LOH	7 (20%)	0.71 (0.45-1.00)	1.32 (0.25-6.89)	
**SMARCA2**				0.55
Norm	23 (65.7%)	0.86 (0.72-1.00)		
Gain	3 (8.6%)	1.00 (1.00-1.00)	Not estimated	
LOSS/LOH	9 (25.7%)	0.71 (0.43-1.00)	1.35 (0.25-7.14)	
**PAX5**				0.59
Norm	24 (68.6%)	0.81 (0.66-1.00)		
Gain	3 (8.6%)	1.00 (1.00-1.00)	Not estimated	
LOSS/LOH	8 (22.8%)	0.88 (0.67-1.00)	0.65 (0.08-5.47)	
**XPA; FOXE1; HEMGN**				
Norm	24 (68.6%)	0.81 (0.65-1.00)		0.59
Gain	3 (8.6%)	1.00 (1.00-1.00)	Not estimated	
LOSS/LOH	8 (22.8%)	0.88 (0.67-1.00)	0.53 (0.06-4.46)	
**USP45**				
Norm	24 (68.6%)	0.77 (0.61-0.97)		0.59
Gain	7 (20%)	1.00 (1.00-1.00)	Not estimated	
LOSS/LOH	4(11.4%)	1.00 (1.00-1.00)	Not estimated	
**NF1**				0.36
Norm	24 (68.6%)	0.86 (0.72-1.00)		
Gain	5 (14.3%)	1.00 (1.00-1.00)	Not estimated	
LOSS/LOH	6 (17.1%)	0.67 (0.38-1.00)	2.31 (0.44-12.26)	
**IGF2BP1**				0.23
Norm	25 (71.4%)	0.86 (0.73-1.00)		
Gain	5 (14.3%)	1.00 (1.00-1.00)	Not estimated	
LOSS/LOH	5 (14.3%)	0.86 (0.73-1.00)	2.95 (0.56-15.60)	
**ERG; TMPRSS2**				0.18
Norm	27 (77.1%)	0.80 (0.65-0.98)		
Gain	8 (22.9%)	1.00 (1.00-1.00)	Not estimated	
**CRLF2**				0.23
Norm	26 (74.3%)	0.77 (0.61-0.97)		
Gain	5 (14.3%)	1.00 (1.00-1.00)	Not estimated	
LOSS/LOH	4 (11.4%)	1.00 (1.00-1.00)	Not estimated	
**FGFR3**				0.08
Norm	26 (74.3%)	0.83 (0.69-1.00)		
Gain	7 (20%)	1.00 (1.00-1.00)	6.0 (0.54-66.14)	
LOSS/LOH	2 (5.7%)	NE (NE-NE)	Not estimated	
**IKZF1**				0.75
Norm	27 (22.9%)	0.86 (0.73-1.00)		
LOSS/LOH	8 (77.1%)	0.75 (0.50-1.00)	1.3 (0.26-6.73)	
**RUNX2**				0.08
Norm	27 (77.1%)	0.88 (0.77-1.00)		
Gain	7 (20%)	1.00 (1.00-1.00)	Not estimated	
LOSS/LOH	1 (2.9%)	1.00 (1.00-1.00)	Not estimated	
**ARID1B**				0.34
Norm	27 (77.1%)	0.79 (0.65-0.97)		
Gain	7 (20%)	1.00 (1.00-1.00)	Not estimated	
LOSS/LOH	1 (2.9%)	1.00 (1.00-1.00)	Not estimated	
**CIP2A**				0.39
Norm	29 (82.9%)	0.87 (0.75-1.00)		
LOSS/LOH	6 (17.4%)	0.67 (0.38-1.00)	2.06 (0.40-10.70)	
**PIK3CA**				0.16
Norm	28 (80%)	0.87 (0.74-1.00)		
Gain	1 (2.9%)	1.00 (1.00-1.00)	Not estimated	
LOSS/LOH	6 (17.1%)	0.67 (0.38-1.00)	3.78 (0.84-17.0)	
**ATM**				0.06
Norm	29 (82.6%)	0.87 (0.75-1.00)		
Gain	3 (8.6%)	0.33 (0.07-1.00)	Not estimated	
LOSS/LOH	3 (8.6%)	1.00 (1.00-1.00)	5.09 (0.97-26.7)	
**RB1**				0.95
Norm	30(85.7%)	0.85 (0.72-1.00)		
LOSS/LOH	5 (14.3%)	0.80 (0.52-1.00)	1.06 (0.13-8.93)	
**BIRC3**				0.06
Norm	29 (82.6%)	0.87 (0.74-1.00)		
Gain	3 (8.6%)	0.33 (0.07-1.00)	Not estimated	
LOSS/LOH	3 (8.6%)	1.00 (1.00-1.00)	5.01 (0.97-26.27)	
**MYC**				0.20
Norm	29 (82.9%)	0.80 (0.66-0.98)		
Gain	6 (17.1%)	1.00 (1.00-1.00)	Not estimated	

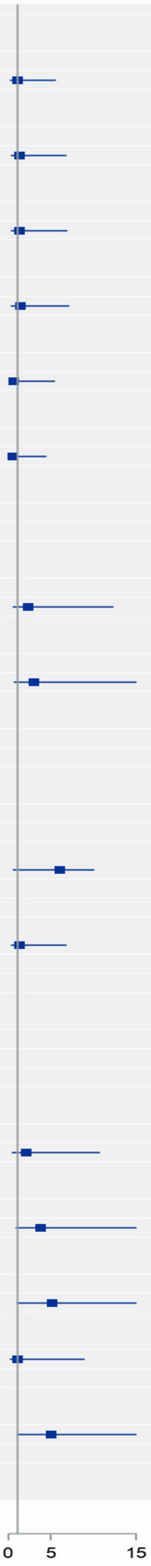

Figure S1. Univariate survival analysis plot of the association of overall survival with the CNA for each gene from the selected panel.
