# Supplementary material for: DNA Copy Number Alterations and Copy Neutral Loss of Heterozygosity in Adult Ph-Negative Acute B-Lymphoblastic Leukemia: Focus on the Genes Involved
Source: Int J Mol Sci. 2023 Dec 18;24(24):17602. doi: 10.3390/ijms242417602 (PMC10744257; doi:10.3390/ijms242417602)
Supplement: Supplementary file 1 [file ijms-24-17602-s001.zip › Table S4.pdf]

Table S4. Aberrations involving the DOCK8 gene identified in the study cohort.

patient#	CN State	Type	Chromosome	Cytoband Start	Cytoband End	Size (kbp)	Marker Count	Gene Count	OMIM Genes Count	OMIM Genes
34	3.0	Gain	9	p24.3	p24.3	68	142	2	**1**	***DOCK8***
6	3.0	Gain	9	p24.3	p24.3	202	395	2	**2**	***DOCK8***, *KANK1*
21	0.7	Loss	9	p24.3	p24.2	4341	3370	18	**12**	***DOCK8***, *KANK1, DMRT1 etc.*
3		cnLOH	9	p24.3	p21.3	21751	6706	110	**80**	***DOCK8, KANK1, DMRT1 etc.***
10		cnLOH	9	p24.3	p13.3	33210	12420	156	**111**	*DOCK8, KANK1, DMRT1 etc.*
25		cnLOH	9	p24.3	p13.2	37064	14068	246	**162**	*DOCK8, KANK1, DMRT1 etc.*
23		cnLOH	9	p24.3	p13.1	38567	14553	262	**171**	*DOCK8, KANK1, DMRT1 etc.*
18	1.69	Loss	9	p24.3	p12	39384	14557	266	**172**	*DOCK8, KANK1, DMRT1 etc.*
39	1.5	Loss	9	p24.3	q34.3	137920	42997	1097	**609**	*DOCK8, KANK1, DMRT1 etc.*
5	1.81	Loss	9	p24.3	q34.3	137920	42997	1097	**609**	*DOCK8, KANK1, DMRT1 etc.*
7	1.79	Loss	9	p24.3	q34.3	137920	42997	1097	**609**	*DOCK8, KANK1, DMRT1 etc.*
15	2.42	Gain	9	p24.3	q34.3	137920	42997	1097	**609**	*DOCK8, KANK1, DMRT1 etc.*
17	1.0	Loss	9	p24.3	q34.3	137920	42997	1097	**609**	*DOCK8, KANK1, DMRT1 etc.*
30	2.19	Gain	9	p24.3	q34.3	137920	42997	1097	**609**	*DOCK8, KANK1, DMRT1 etc.*
